# Supplementary material for: The translational landscape of HIV-1 infected cells reveals key gene regulatory principles
Source: Nat Struct Mol Biol. 2025 Jan 15;32(5):841–52. doi: 10.1038/s41594-024-01468-3 (PMC12086091; doi:10.1038/s41594-024-01468-3)
Supplement: Supplementary file 1 — Supplementary Fig. 1, table legends and Source Data inventory. [file 41594_2024_1468_MOESM1_ESM.pdf]

nature structural & molecular biology

Supplementary information https://doi.org/10.1038/s41594-024-01468-3

The translational landscape of HIV-1 infected cells reveals key gene regulatory principles

In the format provided by the authors and unedited

Supplementary Data

Supplementary Figure 1	*Page 2*
Supplementary tables legends	*Page 3*
Source data inventory	*Page 4*

Supplementary Figure 1

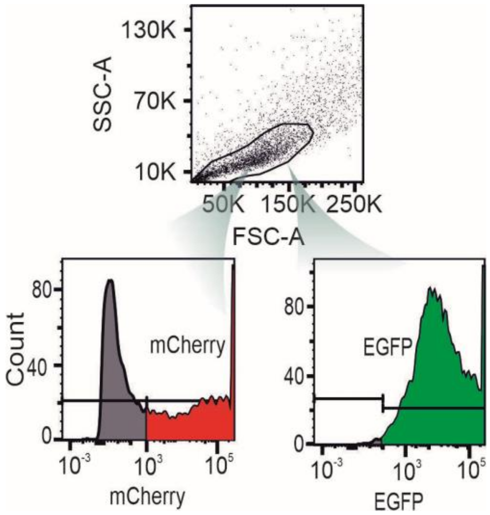

Supp. Figure 1: Gating strategy for flow cytometry determining FE in HEK293 cells. Cell populations were determined based on SSC and FSC and further analyzed for the mean intensities of EGFP and mCherry.

Supplementary Tables

Supp. Table 1: Overview of gene ontology terms associated with differentially regulated genes during HIV-1 infection. For gene ontology (GO) a PANTHER overrepresentation test (Fisher's Exact Test, with FDR correction) for biological processes was performed. The results were obtained with a hierarchical order between GO terms. This table contains the top 15 enriched gene ontology terms in the hierarchy level 1, sorted by false discovery rate (FDR) for the differentially expressed genes with a RiboSeq fold change above- (ribo_up) or below (ribo_down) zero, for each HIV-1 infection time point. The table includes the numbers of genes in our dataset found in the biological process (input_list.number_in_list), the fold enrichment in comparison to the expected number of genes, FDR score, p-value, Gene IDs, the original process as well as the umbrella term.

Supp. Table 2: List of ORFs predicted in the host and virus via PRICE analysis. This table contains the extensive list of open reading frames predicted in both host and viral genomes as characterized by the PRICE bioinformatics pipeline. The table includes columns that specify the unique Gene ID, the ORF ID, ORF location within the genome, the first codon within the ORF, ORF type, and the PRICE specific Start score and range-score. Additional quantitative data provided include the *p*-value (computed using the generalized binomial distribution), the number of mapped reads (fractional for multimapping reads) per experimental condition, and the total number of reads for each ORF.

Supp. Table 3: Riboseq and RNASeq reads mapped to the HIV-1 genome spanning donor splice sites D2 and D3 with different acceptor sites. RiboSeq and RNASeq reads were mapped splice aware with the STAR-aligner to the HIV-1 genome. For stringency, no mismatches in RiboSeq mapping reads were allowed in this analysis. Furthermore, only RiboSeq reads with a read length >= 20 were considered. The reads spanning the D2-AX and D3-AX splice sites were counted and are listed under the respective splice acceptor site. Because ribosome protected fragments are only around 30 nt long, we further filtered for reads having at least 10 nucleotides on either side of the splicing event (min10 columns). Splice sites are characterized by a sudden drop (donor) or increase (acceptor) of read coverage. Therefore, the coverage 1 nt before and 1 nt after the donor sites was investigated. The difference between those coverages allows an estimation of how many splice site overlapping reads are expected.

Supp. Table 4: Proteome analysis of HIV-1 infected cells via mass-spectrometry. This table includes the list of different peptides identified via mass-spectrometry of 24h uninfected and HIV-1- infected cells, analyzed with PEAKS Studio Xpro (Bioinformatics Solutions Inc., Canada).

Supp. Table 5: Nanopore sequencing of HIV-1 transcripts associated with mass spectroscopy detected peptides. This table includes the splice sites, isoforms and the number of corresponding reads found at each time point of infection (n=2). Colors indicate whether the isoform is likely to express one or both of the detected peptides.

Supp. Table 6: List of plasmids and oligos.

Source data inventory

Source Data Fig. 1

Input gene lists and raw output of gene ontology analysis for Fig. 1C.

Source Data Fig. 2

Data for polysome and qRT-PCR profiles for Fig. 2.

Source Data Fig. 6

Data for reporter assays of HIV-1 frameshifting.

Source Data Extended Data Fig. 2

Data for qRT-PCR profiles for ED_ Fig. 2.

Source Data Extended Data Fig. 4

Data for reporter assays of *Vif* iORF translation.

Source Data Extended Data Fig. 6

Uncropped blot of ribosome pausing shown in Fig. 6D.
