# Supplementary material for: The translational landscape of HIV-1 infected cells reveals key gene regulatory principles
Source: Nat Struct Mol Biol. 2025 Jan 15;32(5):841–52. doi: 10.1038/s41594-024-01468-3 (PMC12086091; doi:10.1038/s41594-024-01468-3)
Supplement: Supplementary file 2 — Reporting Summary [file 41594_2024_1468_MOESM2_ESM.pdf]

nature portfolio

Corresponding author(s): Prof. Neva Caliskan

Last updated by author(s): Nov 26, 2024

Reporting Summary

Nature Portfolio wishes to improve the reproducibility of the work that we publish. This form provides structure for consistency and transparency in reporting. For further information on Nature Portfolio policies, see our Editorial Policies and the Editorial Policy Checklist.

Statistics

For all statistical analyses, confirm that the following items are present in the figure legend, table legend, main text, or Methods section.

n/a	Confirmed	
☐	☒	The exact sample size (*n*) for each experimental group/condition, given as a discrete number and unit of measurement
☐	☒	A statement on whether measurements were taken from distinct samples or whether the same sample was measured repeatedly
☐	☒	The statistical test(s) used AND whether they are one- or two-sided<br>*Only common tests should be described solely by name; describe more complex techniques in the Methods section.*
☐	☒	A description of all covariates tested
☐	☒	A description of any assumptions or corrections, such as tests of normality and adjustment for multiple comparisons
☐	☒	A full description of the statistical parameters including central tendency (e.g. means) or other basic estimates (e.g. regression coefficient) AND variation (e.g. standard deviation) or associated estimates of uncertainty (e.g. confidence intervals)
☐	☒	For null hypothesis testing, the test statistic (e.g. *F*, *t*, *r*) with confidence intervals, effect sizes, degrees of freedom and *P* value noted<br>*Give P values as exact values whenever suitable.*
☒	☐	For Bayesian analysis, information on the choice of priors and Markov chain Monte Carlo settings
☒	☐	For hierarchical and complex designs, identification of the appropriate level for tests and full reporting of outcomes
☐	☒	Estimates of effect sizes (e.g. Cohen's *d*, Pearson's *r*), indicating how they were calculated

Our web collection on statistics for biologists contains articles on many of the points above.

Software and code

Policy information about availability of computer code

Data collection	Polysome profiling - monitoring absorbance at 260 and 280 nm TriAX Flow Cell version 1.56A<br>qRT-PCR - CFX Maestro software version 2.3<br>Flow cytometry - NovoCyte Quanteon (ACEA) instrument (Novoexpress (version 1.6.2)).<br>In vitro translation- ImageJ 1.53c<br>Nanopore sequencing - MinKNOW version 23.04.3
Data analysis	Graphpad prism 9 software (version 9.5.1) (https://www.graphpad.com/scientific-software/prism/);<br>Polysome profiles and qRT PCR - Data exported to Excel and visualized in Graphpad.<br>Flow cytometry: Mean fluorescence intensities by Novoexpress software (version 1.6.2) Mean data exported to Excel and FE calculated with Excel - Visualized in Graphpad<br>Riboseq data -PRICE pipeline (https://github.com/erhard-lab/price), DeltaTE pipeline (https://github.com/SGDDNB/translational_regulation).<br>DMS-Map data - RNA Framework v2.7.2, DREEM clustering algorithm (https://codeocean.com/capsule/6175523/tree/v1_), StructureEditor (version 1.0)<br>NanoLC-MS/MS - Peaks Studio Xpro 11<br>Nanopore sequencing - Guppy version 6.5.7, Isoquant version 3.3.0, Biopython 1.79

For manuscripts utilizing custom algorithms or software that are central to the research but not yet described in published literature, software must be made available to editors and reviewers. We strongly encourage code deposition in a community repository (e.g. GitHub). See the Nature Portfolio guidelines for submitting code & software for further information.

Data

Policy information about availability of data

All manuscripts must include a data availability statement. This statement should provide the following information, where applicable:

- Accession codes, unique identifiers, or web links for publicly available datasets
- A description of any restrictions on data availability
- For clinical datasets or third party data, please ensure that the statement adheres to our policy

High-throughput sequencing data have been submitted to Gene Expression Omnibus (GEO) and are available under the accession number GSE244468. The mass spectrometry proteomics data have been deposited to the ProteomeXchange Consortium via the PRIDE partner repository with the dataset identifier PXD058232, which will be made available upon online publication. Further information and requests for resources and reagents should be directed to and will be fulfilled by the lead contact neva.caliskan@helmholtz-hiri.de.

Research involving human participants, their data, or biological material

Policy information about studies with human participants or human data. See also policy information about sex, gender (identity/presentation), and sexual orientation and race, ethnicity and racism.

Reporting on sex and gender	Not applicable
Reporting on race, ethnicity, or other socially relevant groupings	Not applicable
Population characteristics	Not applicable
Recruitment	Not applicable
Ethics oversight	Not applicable

Note that full information on the approval of the study protocol must also be provided in the manuscript.

Field-specific reporting

Please select the one below that is the best fit for your research. If you are not sure, read the appropriate sections before making your selection.

☒ Life sciences ☐ Behavioural & social sciences ☐ Ecological, evolutionary & environmental sciences

For a reference copy of the document with all sections, see nature.com/documents/nr-reporting-summary-flat.pdf

Life sciences study design

All studies must disclose on these points even when the disclosure is negative.

Sample size	No sample-size calculation was done. n=3 independent replicates for all in-vitro or cell line experiments, which is the standard sample size for statistically significant biological tests. For all sequencing experiments, n = 2 independent replicates were made for all high-throughput sequencing experiments, including ribosome and disome profiling and RNA-Seq analyses. This is in line with the standard protocols in the field. This sample size is sufficient for the statistical analysis conducted by PRICE and DeltaTE pipelines. Detailed information about teh sample numbers for each experiment is provided in the manuscript.
Data exclusions	No data was excluded from the analysis.
Replication	The reproducibility of our experiments was confirmed, with 2 replicates tested for deep sequencing experiments. For the in vitro ribosome pausing and dual fluorescence assays were conducted in triplicates. All 3 replicate values are shown on the graph for dual fluorescence reporter assays, and representative of the 3 replicates is shown for the ribosome pausing.
Randomization	Randomization is not relevant to this study because the samples were not allocated into seperate experimental groups.
Blinding	Blinding is not applicable to this study because all data were acquired by machines or by custom/publicly available scripts.

Reporting for specific materials, systems and methods

We require information from authors about some types of materials, experimental systems and methods used in many studies. Here, indicate whether each material, system or method listed is relevant to your study. If you are not sure if a list item applies to your research, read the appropriate section before selecting a response.

Materials & experimental systems

n/a	Involved in the study
☒	☐ Antibodies
☐	☒ Eukaryotic cell lines
☒	☐ Palaeontology and archaeology
☒	☐ Animals and other organisms
☒	☐ Clinical data
☒	☐ Dual use research of concern
☒	☐ Plants

Methods

n/a	Involved in the study
☒	☐ ChIP-seq
☐	☒ Flow cytometry
☒	☐ MRI-based neuroimaging

Eukaryotic cell lines

Policy information about cell lines and Sex and Gender in Research

Cell line source(s)	HEK293T cells (gift from Prof. Jörg Vogel, HIRI-HZI; originally from ATCC), SupT1 cells ((NIH HIV Reagent Program, Division of AIDS, NIAID, NIH: Sup-T1 Cells, ARP-100, contributed by Dr. Dharam Ablashi)
Authentication	Cell lines were not aunthenticated
Mycoplasma contamination	All cell lines negative for mycoplasma contamination (checked via PCR with primers specific for mycoplasma as well as confirmed in RNASeq)
Commonly misidentified lines (See ICLAC register)	No commonly misidentified cell lines used

Plants

Seed stocks	n/a
Novel plant genotypes	n/a
Authentication	n/a

Flow Cytometry

Plots

Confirm that:

☒ The axis labels state the marker and fluorochrome used (e.g. CD4-FITC).

☒ The axis scales are clearly visible. Include numbers along axes only for bottom left plot of group (a 'group' is an analysis of identical markers).

☒ All plots are contour plots with outliers or pseudocolor plots.

☒ A numerical value for number of cells or percentage (with statistics) is provided.

Methodology

Sample preparation	HEK293 cells were transiently transfected using polyethylenimine (PEI) according to manufacturer's instructions using a 1:12 DNA:PEI ratio with either the control construct or the 1FS construct encoding for the dual-fluorescence EGFP-mCherry translation reporter as outlined in Figure 6. Cells were harvested at 24 h post-transfection. After washing with phosphate-buffered saline (PBS).
Instrument	NovoCyte Quanteon (ACEA)
Software	NovoExpress Software (version 1.6.2)
Cell population abundance	Cells were gated for FSC/SSC to ignore cell debris, which were approximately 70-80% of the population, indicating healthy cells. Then the mean GFP and mcherry intensity of these cells were measured.

Gating strategy

Cells were gated for FSC/SSC to capture the bulk of the healthy cell population and and further analyzed for the mean intensities of EGFP (FITC channel) and mCherry (Texas Red channel). Figure exemplifying the gating strategy is now provided as Supplementary Figure 1.

☒ Tick this box to confirm that a figure exemplifying the gating strategy is provided in the Supplementary Information.
