# Supplementary figures and images for: The translational landscape of HIV-1 infected cells reveals key gene regulatory principles
Source: Nat Struct Mol Biol. 2025 Jan 15;32(5):841–52. doi: 10.1038/s41594-024-01468-3 (PMC12086091; doi:10.1038/s41594-024-01468-3)

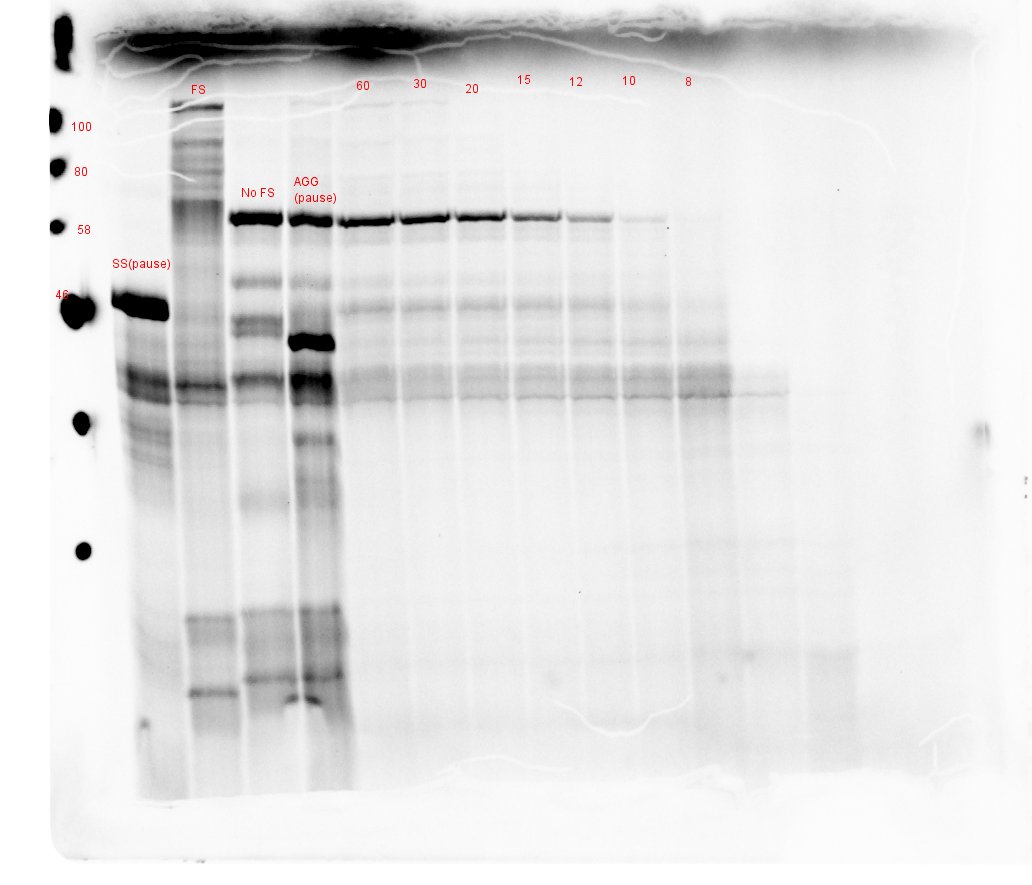

Supplement: Supplementary file 10 — Uncropped blot of ribosome pausing shown in Extended Data Fig. 6d. [file 41594_2024_1468_MOESM10_ESM.jpg]
